# Palygorskite Supported AuPd Alloy Nanoparticles as Efficient Nano-Catalysts for the Reduction of Nitroarenes and Dyes at Room Temperature

**DOI:** 10.3390/nano8121000

**Published:** 2018-12-03

**Authors:** Jun Xu, Shengli Guo, Lei Jia, Wensheng Zhang

**Affiliations:** College of Chemistry and Chemical Engineering, Henan Polytechnic University, Jiaozuo 454000, China; xjjl@hpu.edu.cn (J.X.); guoshenglihpu@163.com (S.G.); zhangwenshenghpu@163.com (W.Z.)

**Keywords:** palygorskite, AuPd alloy, nitroarenes, artificial wastewater

## Abstract

In this work, AuPd alloy palygorskite based Pal-NH_2_@AuPd nano-catalysts were prepared and used as catalysts for the reduction of nitroarenes and dyes at room temperature. The surface of palygorskite (Pal) was first modified with 3-aminpropyltriethoxysilane, and then covered with AuPd alloy nanoparticles through co-reduction of HAuCl_4_ and K_2_PdCl_4_. The morphology and structures of the Pal-NH_2_@AuPd nano-catalysts were characterized by X-ray diffraction (XRD), energy-dispersive X-ray spectroscopy (EDS) and transmission electron microscopy (TEM). The as-synthesized Pal-NH_2_@AuPd nano-catalysts displayed excellent catalytic performance in reducing 4-nitrophenol (4-NP) and various other nitroaromatic compounds. Moreover, the catalytic activities of the Pal-NH_2_@AuPd nano-catalysts were adjustable via changing the atomic ratio of AuPd alloy nanoparticles, leading to the Pal-NH_2_@Au_48_Pd_52_ component as having the best atomic ratio. The Pal-NH_2_@Au_48_Pd_52_ continued to display good catalytic stability after being reused for several cycles and there were no obvious changes, either of the morphology or the particle size distribution of the nano-catalysts. Furthermore, these Pal-NH_2_@Au_48_Pd_52_ nano-catalysts also provided a convenient and accessible way for the degradation of dyes in artificial industrial wastewater.

## 1. Introduction

Nitrobenzene has become extremely widespread in the environment due to the industrial use in the production of dyes, pharmaceuticals, pesticides and explosives [1]. As a hazardous organic compound, nitrobenzene released to the environment can irreversibly damage the health of human and animals [2]. Researchers have reported that the reduction of nitro compounds to amines compounds is highly desirable because organic amines are notable intermediates for many industrial processes, such as in the production of antipyretic drugs, dyes and polymers [3]. Many different methods have been used for the reduction of nitrobenzene or 4-nitrophenol (4-NP) to 4-AP but catalytic reduction of nitrophenol is one the simplest and most environmentally friendly methods using noble metal nanoparticles (NPs). There are many reports about metallic nanoparticles, such as Ag [4], Au [5], Pd [6], Pt [7], and Rh [8], used as catalysts for the reduction reactions.

In recent years, the development of noble metal particles with controlled shapes and sizes has been pursued for attaining desirable catalytic properties [9,10]. In the past decade, noble metal NPs have attracted considerable attention due to their unique physical and chemical properties [11,12,13,14,15,16]. Marco Filice et al. reported heterogeneous enzyme-Pd nanoparticle biohybrids with high catalytic performance in C–C bond formation and tandem catalysis [11]. Le et al. noted that Pd NP supported on nano-silica was utilized for the catalytic reduction of 4-NP [17]. Wang et al. used MOF (metal organic framework) decorated with Pd NP as a catalyst, which showed great catalytic activity for the reduction of 4-NP at room temperature [18]. Bimetallic nanomaterials have recently attracted significant attention due to theoretical and potential applications as a new class of efficient catalysts [19]. In fact, bimetallic nanoparticles have been studied for many applications because their synergistic optical, electronic and catalytic advantages are clearly different from those of their parent metals [19,20]. Palladium-based alloy nanoparticles have been used as catalysts for reactions such as glycerol oxidation [21], CO oxidation [22], and nitroarenes reduction [23]. For example, Shota et al. reported that Pd-Ag alloy NPs had a higher activity for selective oxidation of glycerol to dihydroxyacetone [21]. Hosseini’s group prepared PdCo alloy NPs well-dispersed on polypropylenimine dendrimer–grafted graphene, which can act as a highly efficient catalyst for direct formic acid fuel cells [24]. Other alloy catalysts, such as AuPd alloy, also displayed various catalytic or sensing activities [25,26,27,28]. However, noble metal NPs can easily aggregate due to their high specific surface energy, and their high cost and difficult removal from the reaction media also limit their large-scale applications.

Palygorskite (formula Si_8_O_20_Mg_5_(Al)(OH)_2_(H_2_O)_4_·4H_2_O) is a kind of natural hydrous magnesium–aluminum silicate non-metallic mineral with a special one-dimensional fibrous morphology [29]. Great attention has been paid to the utilization of palygorskite, such as in adsorbents, adhesives, catalysts, and catalyst supports, due to its low-cost, eco-friendliness, unique structure and considerable textual properties [30]. It has a large surface area exhibiting excellent activity. Therefore, palygorskite (Pal) is a good choice as a catalyst support. Wang et al. [24] prepared Pd-Cu/Pal catalysts by an ammonia evaporation method and found that the composite exhibited excellent activity and stability in CO oxidation at room temperature. Liu et al. [29] obtained Pal/Fe-Ni via a liquid-phase reduction method and investigated its degradation of 2,2′,4,4′-tetrabromodiphenylether.

To accomplish the above, in this study we report a simple way to modify Pal with 3-aminopropyltriethoxysilane in toluene and the formation of Au-Pd bimetallic alloy on Pal-NH_2_. By varying the feeding amounts of K_2_PdCl_4_, fine control was obtained over the synthesis of the Au-Pd bimetallic alloy supported on Pal-NH_2_. To investigate the correlations between the nanostructure of the bimetallic and the synergistic effects of the Au and Pd, we produced the Pal-NH_2_@AuPd as a catalyst in the reduction of 4-nitrophenol (4-NP). The as-synthesized Pal-NH_2_@AuPd nano-catalysts with atomic ratio of Au and Pd 48:52 exhibited superior activity compared with other Pal-NH_2_@AuPd nano-catalysts, monometallic Pal-NH_2_@Au and Pal-NH_2_@Pd, owing to the synergistic effect between Au and Pd. More importantly, the Pal-NH_2_@AuPd nanorods displayed excellent activity and chemical stability for long-term catalytic reactions, indicating the Pal-NH_2_@AuPd as a potential effective catalyst. Furthermore, the nano-catalysts reported here also indicated good wastewater treatment ability under alkaline and high salinity conditions. The study presented here provided an impetus for the application of Pal-based composite material in redox catalysis.

## 2. Materials and Methods

### 2.1. Materials and Reagents

Palygorskite was purchased from Jiangsu Autobang Corporation (Huaian, China). Potassium tetrachloropalladate (K_2_PdCl_4_), hydrogen tetrachloroaurate (HAuCl_4_) starch, sucrose, Congo red (CR), reactive red (RR), acetic acid, sulphuric acid, sodium hydroxide, sodium carbonate, sodium chloride and sodium dodecyl sulphate were obtained from Sinopharm Chemistry Reagent Co., Ltd. (Beijing, China). 3-aminpropyltriethoxysilane, 4-nitrophenol, p-nitroaniline, m-nitroaniline, o-Nitroaniline (o-NA), 2,4-Nitroaniline, and sodium hydroxide (NaBH_4_) were bought from Sigma Aldrich (Burlington, NJ, USA). All chemicals were of chemical reagent grade and used without any further purification. Ultrapure water was prepared by using NANO Pure Infinity System (Barnstead/Thermolyne Corp., Dubuque, IA, USA) and was used throughout all the experimental processes.

### 2.2. Preparation of Pal-NH_2_

In order to introduce terminal amino groups onto the palygorskite surface [31], 500 mg of palygorskite was dispersed into 60 mL toluene with ultrasound for 10 min and then 1 mL of 3-aminopropyltrimethoxysilane was added dropwise to the above mixture with vigorous stirring. After the mixture was reacted for 8 h at temperatures of 90 °C, the solution was continuously stirred for another 1 h at room temperature. The final solution was centrifuged at 5000 rpm for 5 min and the resulting Pal-NH_2_ nanorods were washed with ethanol to remove unreacted material, and dried in vacuum at 60 °C for 2 h. The product was stored in sealed bottle for later use.

### 2.3. Preparation of Pal-NH_2_@AuPd, Pal-NH_2_@Au, Pal-NH_2_@Pd

The concentrations of both HAuCl_4_ and K_2_PdCl_4_ were fixed at 10 mM during the whole preparation. The typical procedure for synthesis of Pal-NH_2_@Au_48_Pd_52_ is as follows. A quantity of 50 mg of Pal-NH_2_ was dispersed in 5.0 mL aqueous solution via ultra-sonication and then 0.48 mL HAuCl_4_ and 0.52 mL K_2_PdCl_4_ were gradually added to the above solution. The resulting reaction mixture was kept in a glass bottle under stirring for 3 h (3000 rpm). Then freshly prepared 1.0 mL NaBH_4_ aqueous solution (28 mg, 0.7 mmol) was added and the reaction mixture was maintained at room temperature for another 0.5 h. Then, the resulting solution was centrifuged (5000 rpm, 5 min) and washed with pure water three times (5 min each time) to remove the remaining reagents and then dried in vacuum at 60 °C for 2 h. For comparison, Pal-NH_2_@Au_33_Pd_67_ (0.33 mL HAuCl_4_ and 0.67 mL K_2_PdCl_4_) and Pal-NH_2_@Au_81_Pd_19_ (0.81 mL HAuCl_4_ and 0.19 mL K_2_PdCl_4_) were also prepared using the same method by adjusting the molar ratios of Au/Pd precursors. Only 1 mL HAuCl_4_ and 1 mL K_2_PdCl_4_ were used for the preparation of Pal-NH_2_@Au and Pal-NH_2_@Pd, respectively.

### 2.4. Catalytic Reduction of Nitrobenzene

In order to examine the catalytic activity of the Pal-NH_2_@Au_48_Pd_52_ nano-catalysts, we chose the reduction of 4-NP with the help of NaBH_4_ at room temperature as a model reaction. In a typical procedure, 0.25 mL freshly prepared NaBH_4_ solution (1.2 M) was mixed with 0.25 mL of 4-NP (3.4 × 10^−3^ M) and 8 mL of deionized water in a glass bottle, followed by addition of 0.02 mL Pal-NH_2_@Au_48_Pd_52_ solution (1 mg/mL) to the mixture. The color of the solution gradually vanished as the reaction proceeded. The progress of the reaction solution was immediately recorded using UV-Vis spectra at 1 min intervals. The catalytic reductions of other nitrobenzenes were conducted under the same conditions as for 4-NP. To evaluate the reusability of catalyst, the catalysts were collected by centrifugation (8000 rpm, 3 min), washed with pure water, dried at 60 °C and reused for reduction under the same conditions in the next cycles.

### 2.5. Preparation of Artificial Wastewater

The artificial wastewater was prepared as in the reported procedure [32]. Artificial wastewater was prepared by mixing starch (25 mg/L), sucrose (15 mg/L), CR (2.5 mg/L), RR (2.5 mg/L), acetic acid (5 mg/L), sulphuric acid (7.5 mg/L), sodium hydroxide (12.5 mg/L), sodium carbonate (12.5 mg/L), sodium chloride (75 mg/L), and sodium dodecyl sulphate (2.75 mg/L) in a 1000 mL volumetric flask. The pH of the resulting solution was 9.5.

### 2.6. Treatment of Artificial Wastewater with Pal-NH_2_@Au_48_Pd_52_

A quantity of 3 mL of artificial wastewater was decolorized by adding 40 μg Pal-NH_2_@Au_48_Pd_52_ and 100 μL of 50 mM NaBH_4_. The reaction progress was monitored by ultraviolet–visible (UV-Vis) spectrometry at a certain time interval to obtain successive information.

### 2.7. Characterization

Transmission electron microscopy (TEM), high resolution transmission electron microscopy (HRTEM), and the energy dispersive spectra (EDS) were determined using a Tecnai-G2-F30 (FEI, Eindhoven, The Netherlands) at acceleration voltages of 200 kV. Powder X-ray diffraction patterns (PXRD) were recorded on a Rigaku-Dmax 2400 diffractometer using Cu Kα radiation (Rigaku Dmax-2400, Rigaku Corporation, Tokyo, Japan). UV-vis absorption spectra were investigated using a Perkin Elmer Lambda 950 spectrophotometer (Lambda950, Perkin-Elmer, Waltham, MA, USA). The content of Au and Pd was determined by inductively coupled plasma-atomic emission spectroscopy (ICP-AES, ICPE-9800, Shimadzu Corporation, Kyoto, Japan).

## 3. Results and Discussion

### 3.1. Structural and Morphology Characterization

**Figure 1:**
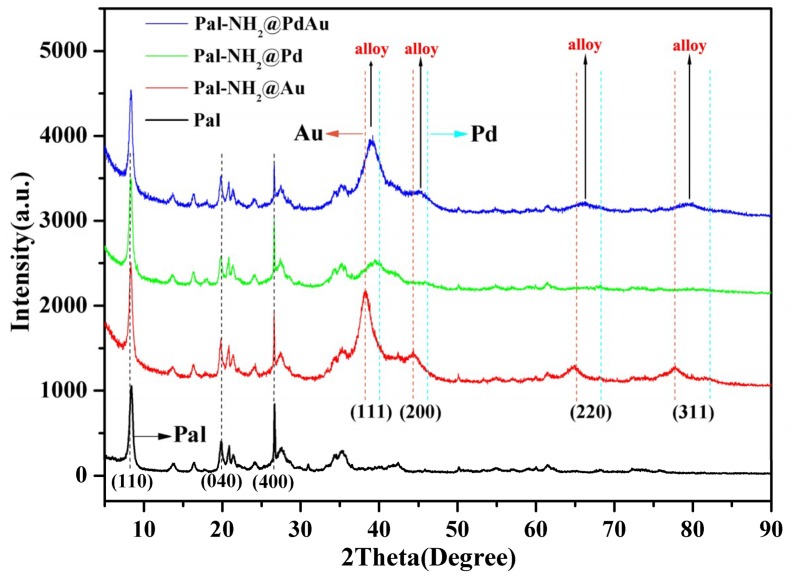
X-ray diffraction (XRD) patterns of Pal, Pal-NH_2_@Au, Pal-NH_2_@Pd and Pal-NH_2_@AuPd.

X-ray diffraction (XRD) patterns were used to characterize the alloy Pal-NH_2_@Au_48_Pd_52_ structure. Figure 1 shows the XRD patterns of Pal, Pal-NH_2_@Au, Pal-NH_2_@Pd, and Pal-NH_2_@Au_48_Pd_52_. The weak and broad diffraction peaks at 20 = 8.3°, 19.7°, and 26.6° were assigned to the diffraction of (110), (040) and (231) planes of palygorskite (JCPDS No. 31-0783) [33] for each sample. The results clearly indicated that the two-plane characteristic peaks for Pal and Pal-NH_2_ did not change, which demonstrated that the original inner structure of palygorskite was not damaged during the whole preparation of Pal-NH_2_@Au_48_Pd_52_. The peaks at 2θ values of 40.4°, 46.5°, 68.4° and 82.1°corresponded to the (111), (200), (220) and (311) planes of face-centered cubic structures of the Pd (JCPDS No. 65-2867) [34]. Pal-NH_2_@Au exhibited four characteristic peaks at 2θ of 38.2°, 44.6°, 64.6° and 77.5°, consistent with the lattice structure of Au (JCPDS No. 65-8601) [35]. Although the Pal-NH_2_-supported Au_48_Pd_52_ alloy nano-catalysts also exhibited four peaks at 2θ of 39.3°, 45.5°, 65.8° and 79.5°, these peaks lay between monometallic Au and Pd peaks. Thus, we could demonstrate that Pd and Au atoms had penetrated into each other’s mutual lattice and formed a AuPd bimetallic alloy structure [36,37].

**Figure 2:**
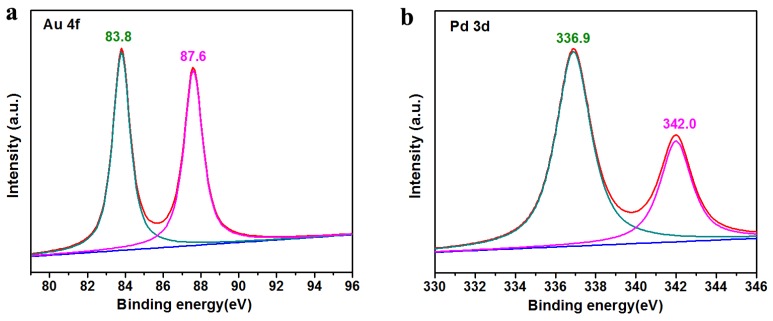
X-ray photoelectron spectroscopy (XPS) spectra of the Pal-NH_2_@Au_48_Pd_52_ sample: (**a**) Au 4f and (**b**) Pd 3d.

In order to further investigate the surface states of AuPd bimetallic alloy NPs on the surface of Atta, the Pal-NH_2_@Au_48_Pd_52_ sample was examined by high-resolution X-ray photoelectron spectroscopy (XPS) analysis of Au 4f and Pd 3d. As shown in Figure 2a, the Au 4f_7/2_ and Au 4f_5/2_ peaks of Pal-NH_2_@Au_48_Pd_52_ alloy located at 83.8 and 87.6 eV can be attributed to metallic Au^0^, which exhibits a slight downshift compared to that of monometallic Au nanoparticles [38]. Furthermore, the Pd 3d spectra (Figure 2b) of the AuPd bimetallic alloy sample could be fitted into two peaks (336.9 and 342.0 eV), which can be attributed to 3d_5/2_ and 3d_3/2_ of Pd^0^, respectively. In addition, the XPS signals of Pd 3d showed a greater binding energy than that of monometallic Pd-doped nanoparticles [38,39]. The above changes in binding energy were attributed to the formation of AuPd bimetallic alloy [25,40,41], which was in good agreement with XRD characterization.

**Figure 3:**
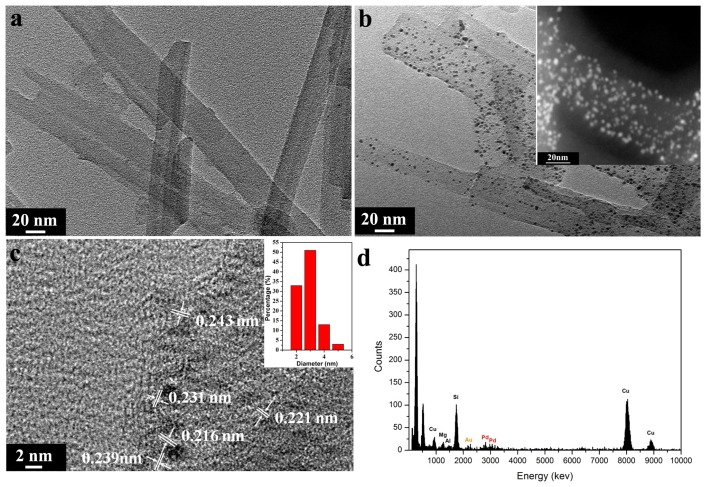
The transmission electron microscopy (TEM) images of the Pal-NH_2_ (**a**) and Pal-NH_2_@Au_48_Pd_52_ (**b**,**c**), and the energy-dispersive X-ray spectroscopy (EDX) spectrum of Pal-NH_2_@Au_48_Pd_52_ nano-catalysts (**d**); The top right corner of **b** is the high angle annular dark-field scanning transmission electron microscopy (HAADF-STEM) images of Pal-NH_2_@Au_48_Pd_52_.

The morphologies of Pal-NH_2_ and Pal-NH_2_@Au_48_Pd_52_ were characterized by transmission electron microscopy (TEM), as shown in Figure 3a–c, indicating that the palygorskite continued to maintain the rod-like morphology after surface modification. This special rod-like morphology also made it possible for it to act as a carrier, allowing AuPd bimetallic nanoparticles to be modified onto the surface of Pal by the simple nucleation and co-precipitation process. From Figure 3b we can see that many small nanoparticles appeared on Pal surface, demonstrating the successful loading of the AuPd alloy nano-catalyst without obvious aggregation. The white spots and gray shell on the high angle annular dark-field scanning transmission electron microscopy (HAADF-STEM) image in the top right corner of Figure 3b also illustrate that the AuPd alloy nano-catalysts were modified on the surface of Pal; the average size of AuPd nano-catalysts was about 3.1 nm. Furthermore, the typical High Resolution Transmission Electron Microscope (HRTEM) image (Figure 3c) showed the arrangement of the AuPd NPs, where AuPd NPs were seen as having various orientations and lattice spacing. By carefully measuring the lattice parameters using a digital micrograph and comparing them with the data in Joint Committee on Powder Diffraction Standards (JCPDS), five different kinds of lattice fringes were clearly observed. However, no diffraction peaks corresponding to Au or Pd NPs can be observed in Figure 3c. Therefore, it was deduced that the remaining intervals belong to AuPd alloys. Meanwhile, the energy-dispersive X-ray spectroscopy (EDX) spectrum of the Pal-NH_2_@AuPd (Figure 3d) exhibited the presence of Al, Si, Mg, Au, and Pd elements, which also proved the successful deposition of AuPd on the surface of Pal-NH_2_. Inductively coupled plasma-atomic emission spectroscopy (ICP-AES) was used to determine the Au or Pd contents (as shown in Table 1) of the original Pal-NH_2_, which were consistent with the designed ratio.

**Figure 4:**
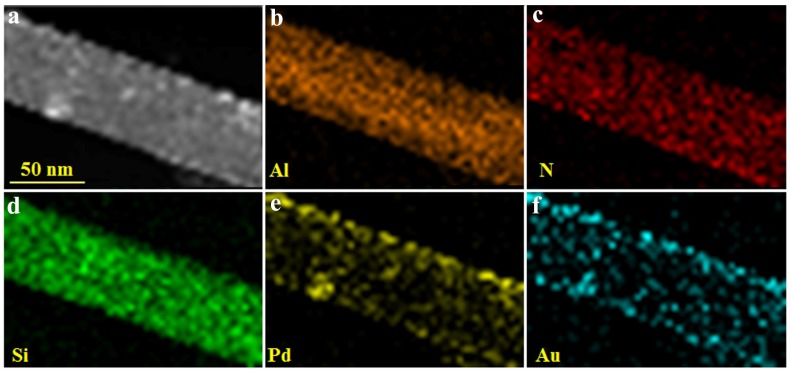
HAADF-STEM images of Pal-NH_2_@Au_48_Pd_52_ (**a**); EDX mapping: of Al element (**b**); N element (**c**); Si element (**d**); Pd element (**e**) and Au element (**f**).

The distribution of each element on the surface of Pal-NH_2_@Au_48_Pd_52_ were further investigated by EDS-mapping and EDX. High angle annular dark-field scanning transmission electron microscopy (HAADF-STEM) measurements (Figure 4) were used to study the stereo distribution of Pd and Au. Here, it is necessary to mention that it was difficult to obtain HAADF-STEM results for the ultrafine AuPd NPs and it is normal when the nanoparticles had small size, organic liagnds and heavy atoms. During the operation of the HAADF-STEM we can’t focus the light on all of the nanoparticles, when the focus and exposure time was too long, carbonization would happen on some of the NPs, which may reduce the clarity of the picture. The dark-field images indicated an abundant deposition of AuPd nano-catalysts on the Pal-NH_2_ support. Clearly from Figure 4, Si, Al, N, Au, and Pd atoms were distributed on Pal-NH_2_, while Pd and Au atoms were distributed uniformly over the entire Pal-NH_2_@Au_48_Pd_52_ nano-catalysts, which provided strong evidence regarding the formation of alloy-decorated nano-catalysts [37].

### 3.2. Catalytic Activity

**Figure 5:**
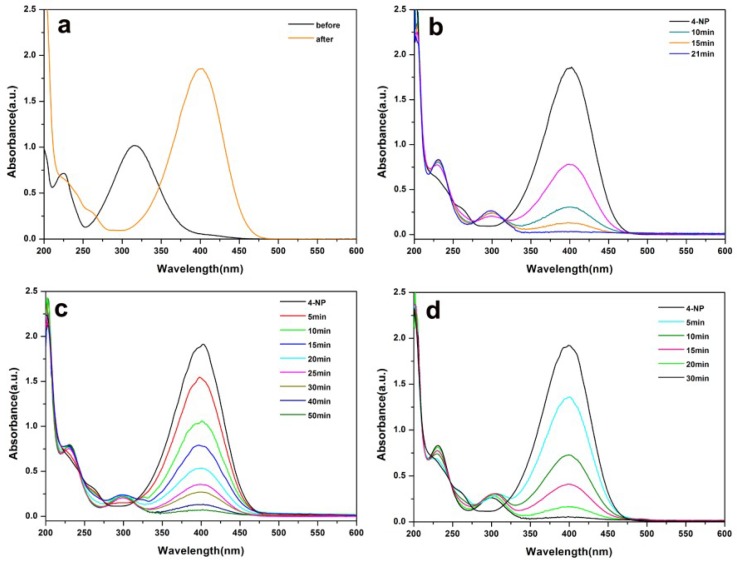
(**a**) UV-Vis absorption spectra of 4-NP before and after the addition of NaBH_4_ solution without catalyst: variation in UV-Vis spectra intervals for the 4-nitrophenol (4-NP) reduction in the presence of (**b**) Pal-NH_2_@Au_48_Pd_52_; (**c**) Pal-NH_2_@Au; and (**d**) Pal-NH_2_@Pd.

Inspired by the attractive properties of the AuPd alloy nanoparticles on Pal, such as their monodisperse, ultrafine and pristine nature, we investigated the catalytic ability of the as-prepared Pal-NH_2_@AuPd. The reduction of 4-NP to 4-aminophenol (4-AP) was carried out as a test reaction using NaBH_4_ as the reducing agent and Pal-NH_2_@AuPd as the catalyst. UV-Vis absorption spectra were used to illustrate the reduction process of 4-NP. Generally, the solution of 4-NP had a distinct absorption peak at 317 nm. However, after the addition of an aqueous solution of NaBH_4_, this peak was remarkably red-shifted to 400 nm owing to the formation of 4-nitrophenolate [42], as shown in Figure 5a. The process was also accompanied by a color change from light yellow to bright yellow. With the addition of Pal-NH_2_@Au_48_Pd_52_ catalyst, the absorption peak at 400 nm gradually disappeared and a new peak appeared at 300 nm, which corresponded to 4-AP [43], as shown in Figure 5b. Meanwhile, the bright yellow solution became colorless over time, which indicated that the reduction of 4-NP was complete. Furthermore, as shown in Figure 5c,d, it was noticed that the absorption intensities at 400 nm of the Pal-NH_2_@Au_48_Pd_52_ nano-catalysts decreased much faster in comparison to Pal-NH_2_@Au and Pal-NH_2_@Pd, which indicated that Pal-NH_2_@Au_48_Pd_52_ nano-catalysts had better catalytic activity. As to the possible catalytic mechanism of this reduction, the Pal-NH_2_@Au_48_Pd_52_ may serve as an electron relay system and play a significant role in the electron transfer (ET) process. The 4-NP molecule and BH_4_^−^ ion were first adsorbed to the surface of the Pal-NH_2_@AuPd and the large specific surface area of the Pal-NH_2_@AuPd catalysts resulted in the concentration increase near the surface of the Pal-NH_2_@AuPd. Then, ET from BH_4_^−^ to the 4-NP via the Pal-NH_2_@AuPd became easier and directly accelerated the reduction reaction rate [44].

**Figure 6:**
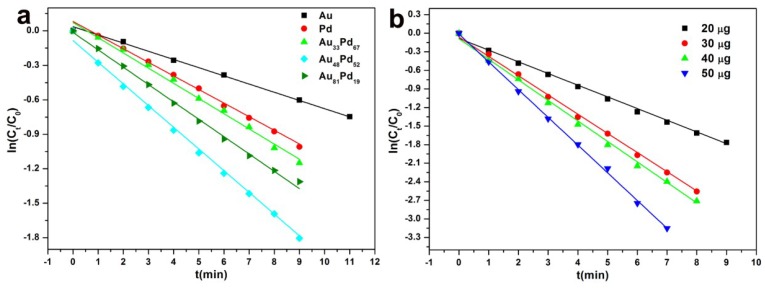
(**a**) Plot of ln(*C_t_*/*C*_0_) versus the reaction time for the reduction of 4-NP over different samples at 25 °C; (**b**) Plot of ln(*C_t_*/*C*_0_) versus the reaction time for the reduction of 4-NP over different amounts of Pal-NH_2_@Au_48_Pd_52_.

Since the amount of NaBH_4_ used was in excess compared to 4-NP, the concentration of BH_4_^−^ remained almost constant throughout the reaction progress. In this way, a pseudo first-order kinetics equation with respect to the 4-NP was applied to calculate the apparent rate constant (*K*_app_), as follows: *k*_app_*t* = ln(*C_t_*/*C*_0_). *C_t_* and *C*_0_ represented the concentrations of 4-NP at intervals and the initial stage, respectively. Figure 6a exhibits the linear plots of ln(*C_t_*/*C*_0_) versus reaction time for the reaction catalyzed by Pal-NH_2_@Au_48_Pd_52_ and supported monometallic samples. The apparent rate constants were 0.194, 0.021, and 0.071 min^−1^ for the Pal-NH_2_@Au_48_Pd_52_, Pal-NH_2_@Au, and Pal-NH_2_@Pd, respectively, demonstrating that the catalytic activity of the AuPd alloy nano-catalysts were roughly 9 and 2.7 times higher than that of their monometallic counterparts. In order to further investigate the effect of the catalytic performance of the alloy NPs, the Pal-NH_2_@AuPd with different Au/Pd ratios were prepared by varying the feeding amounts of K_2_PdCl_4_. The plot of ln(*C_t_*/*C*_0_) versus reaction time was also observed for the Pal-NH_2_@AuPd with different Au/Pd ratios catalyzing under the same condition. As recognized from the change in the rate constants and turnover frequency (TOF) values with the Au and Pd mole fraction (Figure 6a and Table 1), we could conclude that the catalytic property was directly dependent upon the composition of the AuPd alloy NPs, and that the Pal-NH_2_@Au_48_Pd_52_ exhibited the best catalytic activity. This super-catalytic activity of Pal-NH_2_@Au_48_Pd_52_ bimetallic nano-catalysts may be due to the synergistic effect between Au and Pd NPs, as evidenced by extra charge interaction between Pd and Au NPs in Pal-NH_2_@Au_48_Pd_52_. Furthermore, the TEM images of the Pal-NH_2_@Au, Pal-NH_2_@Pd, Pal-NH_2_@Au_33_Pd_67_ and Pal-NH_2_@Au_81_Pd_19_ are also shown in Figure S1. We can see that the sizes of Au or Pd NPs on Pal-NH_2_ surface were smaller than that of Au_33_Pd_67_ or Au_81_Pd_19_ alloy. In spite of the smaller monometallic catalysts, the Au_48_Pd_52_ alloy catalysts still showed the best catalytic activities, which is due to the synergistic effect between Au and Pd NPs, as evidenced by extra charge interaction between Pd and Au NPs in Pal-NH_2_@Au_48_Pd_52_.

In order to study the effect of catalyst loading on 4-NP degradation, the amount of Pal-NH_2_@Au_48_Pd_52_ (20, 30, 40 and 50 μg) was varied while maintaining fixed concentrations of 4-NP and NaBH_4_. The results obtained for quantitative analysis of the nano-catalysts for the 4-NP reduction as a function of time is shown in Figure 6b. It was observed that with an increasing the amount of the nano-catalysts, the reduction ability of 4-NP was enhanced rapidly and it was difficult to observe the progress of the reaction; this can be understood because the increase of catalyst dosage will increase the total number of active sites on the surface of Pal-NH_2_@Au_48_Pd_52_. Hence, the minimum amount of 20 μg catalyst was taken as an optimal catalyst concentration in this research.

In order to compare the catalytic activity of Pal-NH_2_@Au_48_Pd_52_ with other reported catalytic examples containing AuPd NPs in previous literature, we also compared reaction rate constant (*K*) values according to the reported parameter *K* = *k*_app_/*N* [45], where *N* represents the molar ratio of Au-Pd alloy NPs (*n*_Au_ + *n*_Pd_) and 4-NP (*n*_4-NP_). It is well known that the rate constant is influenced by many factors, such as metal nanoparticle loading, the usage of NaBH_4_, and catalysts dosage. In order to compare our results with other reports, we also listed the ratio of apparent rate constants (*k*_app_) over the molar ratio of AuPd catalysts and the concentration of 4-NP, named activity factor *K*, summarized in Table 2. It is worth noting that the activity factor *K* value of Pal-NH_2_@Au_48_Pd_52_ was 552.7 times higher than in Au_1_Pd_4_ core-shell nano-catalysts [46] and 5.6–116.2 times higher than in other reported 4-NP catalytic reactions [47,48,49]. In fact, in our catalytic system, each catalyst could achieve nearly 99% conversion of the substrates, so the ratio of Au-Pd alloy NPs (*n*_Au_ + *n*_Pd_) and 4-NP (*n*_4-NP_) is 1/TON (where TON represents turnover number). Much of the literature adopted the apparent rate constant *k*_app_ to express the speed of a reaction without considering the amount of catalyst used or the concentration of the substrates. If the amount of catalyst and the concentration of the substrates were not considered, the increase in the amount of the catalyst also caused an increase in the *k*_app_ value. Similarly, the substrate concentration decreased and the reaction rate also decreased. Therefore, the calculation of the rate constant K must take into account the above two factors. In our catalytic reaction, the conversion rate of the substrate could reach 99%, so we believed that the concentration of the substrate was the same as the concentration of the product. In order to thoroughly compare the catalytic efficiency of Pal-NH_2_@Au_48_Pd_52_ nano-catalysts with other examples in the literature, we also calculated the TON and TOF values. As shown in Table 2, it was worth noting that the *K*, TON and TOF values of Pal-NH_2_@Au_48_Pd_52_ were higher than for many other nano-catalysts [44,45,46,47].

**Figure 7:**
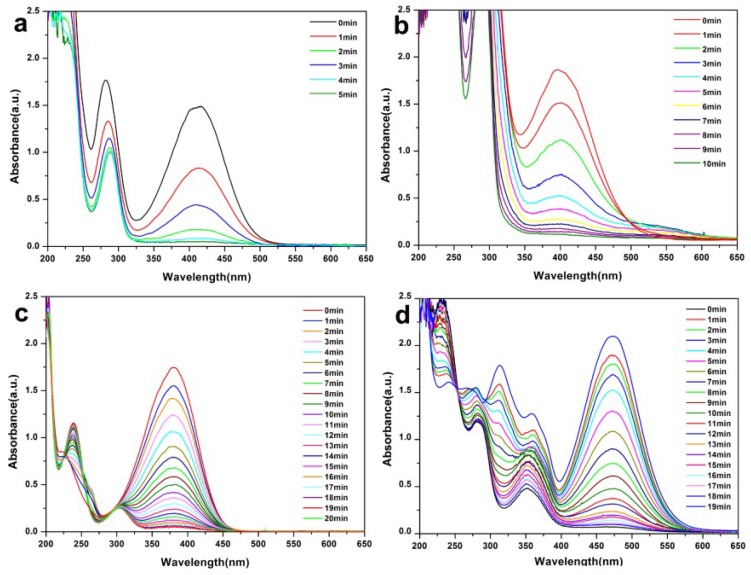
UV-Vis absorption spectra for the reduction of (**a**) o-Nitroaniline; (**b**) m-Nitroaniline; (**c**) p-Nitroaniline; (**d**) 2,4-Nitroaniline using Pal-NH_2_@Au_48_Pd_52_ nano-catalysts.

Furthermore, we investigated the catalytic activity of the Pal-NH_2_@Au_48_Pd_52_ catalyst for the reduction of other four other kinds of nitroaniline nitrobenzene analogs, namely, p-Nitroaniline, m-Nitroaniline, o-Nitroanilineand, and 2,4-Nitroaniline (Table 3). The conversion was determined by the gas chromatography/mass spectroscopy (GC/MS) analysis of the residual nitrobenzene in the mixture after reaction. All the catalytic reaction progresses of other nitrobenzenes were conducted under the same conditions of 4-NP. As shown in Table 2, our catalyst displayed excellent catalytic behavior with excellent yields toward a series of nitrobenzene compounds regardless of different substituent groups. From Figure 7a–d we can see the typical change of UV-Vis spectra of each compound during the reaction clearly demonstrated that the reduction of these nitrobenzenes did occur in the presence of a small amount of Pal-NH_2_@Au_48_Pd_52_. The highest characteristic absorption peak of each substituent gradually weakened within 5–19 min, which also proved the excellent percentage conversion of the prepared Pal-NH_2_@Au_48_Pd_52_ nano-catalysts.

**Figure 8:**
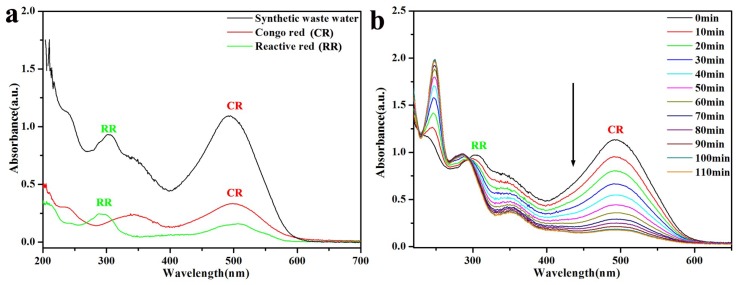
(**a**) The same concentration of artificial wastewater, using Congo red and reactive red; (**b**) artificial wastewater after the reduction by NaBH_4_ in the presence of Pal-NH_2_@Au_48_Pd_52_.

We also extended the application to investigate the catalytic activity of the Pal-NH_2_@Au_48_Pd_52_ nano-catalysts for the treatment of artificial wastewater [32]. From Figure 8a we can see that the typical absorption peaks of the organic dyes Congo red (CR) and reactive red (RR) in artificial wastewater appeared on the ultraviolet spectrum, demonstrating the main absorption peaks were 495 and 305 nm. The two peaks of CR and RR showed a decreasing trend during the 110 min catalytic time, which indicated that the Pal-NH_2_@Au_48_Pd_52_ nano-catalysts prepared also exhibited strong wastewater treatment ability under alkaline and high salinity conditions (Figure 8b). The conversion of the two organic dyes was more than 95% and the color of the artificial wastewater changed from red to colorless. This experiment provided evidence of a convenient and accessible means of degrading dyes in industrial wastewater, and we will further study dye degradation efficiency and the mechanism of the catalytic system in complex industrial wastewater in subsequent research. 

**Figure 9:**
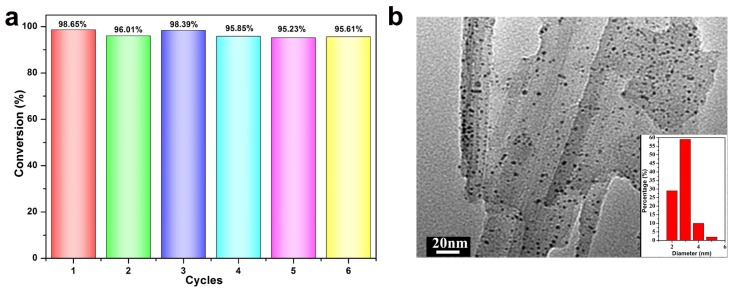
(**a**) The reusability test of Pal-NH_2_@Au_48_Pd_52_ catalyst in five cycles; (**b**) the morphology and the particle size distribution (inset) of the Pal-NH_2_@Au_48_Pd_52_ catalyst after successive cycles.

The reusability of the nanocomposite catalysts is an important feature to be strongly considered in practical heterogeneous catalytic applications. In this study, the reusability of the Pal-NH_2_@Au_48_Pd_52_ was evaluated as an example, by conducting the reduction of 4-NP for six cycles. As illustrated in Figure 9a, the Pal-NH_2_@Au_48_Pd_52_ catalyst displayed good catalytic activity after five cycles without any significant loss of activity and selectivity. In the fifth run, the conversion yield of 4-NP was as high as 96%, indicating the excellent recyclability of the Pal-NH_2_@Au_48_Pd_52_ catalyst. To further confirm the stability of the Pal-NH_2_@Au_48_Pd_52_ nano-catalysts, the sample was collected after five successive catalytic runs and the structure was examined by TEM measurement. From the TEM image in Figure 9b we can clearly see that there were no obvious changes, either in the morphology or the particle size distribution, as compared to the original nano-catalyst. This high recycling stability of the prepared Pal-NH_2_@Au_48_Pd_52_ nano-catalyst indicated that it can be a material of choice for the reduction of 4-NP.

## 4. Conclusions

In conclusion, we reported an effective and stable noble Pal-NH_2_@AuPd alloy nano-catalyst for the reduction of 4-NP with NaBH_4_ under mild reaction conditions. The activity of bimetallic AuPd nanoparticles can be greatly enhanced after adjusting the suitable molar ratio of Au and Pd atoms. Compared to the original monometallic nanocomposite (Pal-NH_2_@Au and Pal-NH_2_@Pd), the alloy Pal-NH_2_@Au_48_Pd_52_ indicated excellent catalytic activities toward the reduction of 4-nitrophenol. This method, used for the decoration of bimetallic nano-catlysts, was simple and versatile, and no stabilizer was necessary for the growth of the bimetallic nanoparticles, which is of considerable technological importance. Furthermore, Pal-NH_2_@Au_48_Pd_52_ also indicated excellent activities in the reduction of other nitroarenes and dyes in artificial wastewater, regardless of the alkaline and high salinity conditions. Thus, as a fascinating rod-like structure, amine groups-modified palygorskite can be potentially used as a novel catalyst to support bimetallic AuPd hybrids or other metal catalysts.

## Figures and Tables

**Table 1:** Summary of the molar ratios between Au and Pd, the weight percentage of Au and Pd (based on ICP-MS results), the rate constants of the reaction (k), and the turnover frequency (TOF) values.

Catalysts	Au (wt%)	Pd (wt%)	K (Min^−1^)	TOF (Min^−1^)
Au	3.94	0	0.021	4.25
Pd	0	2.12	0.071	7.08
Au_33_Pd_67_	1.32	1.41	0.132	12.39
Au_48_Pd_52_	1.81	1.10	0.194	16.51
Au_81_Pd_19_	3.19	0.40	0.102	10.11

**Table 2:** Comparison of the activity of our catalysts with other reported catalysts in terms of the activity factor *K* values and apparent rate constants.

Catalysts	*k*_app_ (min^−1^)	(*n*_Au_ + *n*_Pd_)/*n*_4-NP_	Turnover Number (TON)	*K* (min^−1^)	TOF (min^−1^)	Reference
Pal-NH_2_@Au_48_Pd_52_	0.194	0.0047	212.77	45.116	11.82	Here
Au_1_Pd_4_ core/shell	0.39	15.00	0.037	0.078	3	[43]
Pd@Au core-shell nanotetrapods	0.139	0.380	2.63	0.366	0.035	[44]
Melamine cyanurate-Pd/Au	0.280	0.0364	27.41	7.692	0.036	[45]
Au-on-Pd heteronanostructure	0.867	0.16	33.33	5.419	9.52	[46]

**Table 3:** Reduction of various nitrobenzenes using Pal-NH_2_@Au_48_Pd_52_ (Reaction condition: 0.25 mL of 3.4 × 10^−3^ M substrates and 0.25 mL of 1.2 M fresh NaBH_4_ at the room temperature).

Compound	Time/min	Conversion/%	Amount of Catalyst/μg	TOF (min^−1^)
p-Nitroaniline	20	99	20	9.88
m-Nitroaniline	10	99	20	19.77
o-Nitroaniline	5	99	20	39.54
2,4-Nitroaniline	19	99	20	10.40
m-Nitrotoluene	74	79	20	2.11
o-Nitrotoluene	86	85	20	1.95
2,4-Dinitrotoluene	97	89	20	1.81

## Supplementary Materials

The following are available online at http://www.mdpi.com/2079-4991/8/12/1000/s1, Figure S1: TEM images of Pal-NH_2_@Au (**a**); Pal-NH_2_@Pd (**b**); Pal-NH_2_@Au_33_Pd_67_ (**c**); and Pal-NH_2_@Au_81_Pd_19_ (**d**).
