# Supplementary material for: Palygorskite Supported AuPd Alloy Nanoparticles as Efficient Nano-Catalysts for the Reduction of Nitroarenes and Dyes at Room Temperature
Source: Nanomaterials (Basel). 2018 Dec 3;8(12):1000. doi: 10.3390/nano8121000 (PMC6315398; doi:10.3390/nano8121000)
Supplement: Supplementary file 1 [file nanomaterials-08-01000-s001.pdf]

*Supplementary*

# Palygorskite Supported AuPd Alloy Nanoparticles as Efficient Nano-Catalysts for the Reduction of Nitroarenes and Dyes at Room Temperature

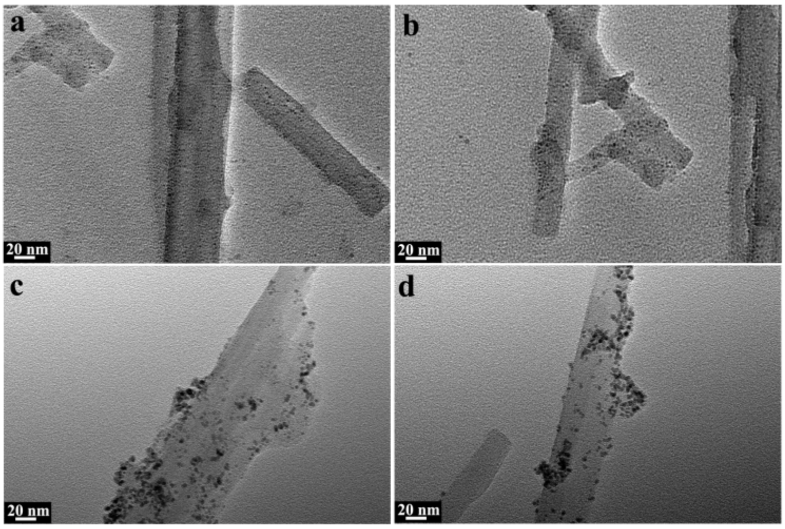


**Figure S1.** TEM images of Pal-$NH_2$@Au (**a**); Pal-$NH_2$@Pd (**b**); Pal-$NH_2$@$Au_{33}Pd_{67}$ (**c**); and Pal-$NH_2$@$Au_{81}Pd_{19}$ (**d**).
